# Ecological Importance of Viral Lysis as a Loss Factor of Phytoplankton in the Amundsen Sea

**DOI:** 10.3390/microorganisms10101967

**Published:** 2022-10-05

**Authors:** Charlotte Eich, Tristan E. G. Biggs, Willem H. van de Poll, Mathijs van Manen, Hung-An Tian, Jinyoung Jung, Youngju Lee, Rob Middag, Corina P. D. Brussaard

**Affiliations:** 1 Department of Marine Microbiology and Biogeochemistry, NIOZ Royal Netherlands Institute for Sea Research, 1797 SZ ’t Horntje, The Netherlands; 2 Institute for Biodiversity and Ecosystem Dynamics (IBED), University of Amsterdam, 1098 XH Amsterdam, The Netherlands; 3 RUG Rijksuniversiteit Groningen, Faculty of Science and Engineering, 9712 CP Groningen, The Netherlands; 4 Department of Ocean Systems, NIOZ Royal Netherlands Institute for Sea Research, 1797 SZ ’t Horntje, The Netherlands; 5 Centre for Isotope Research-Oceans, Energy and Sustainability Research Institute Groningen, Faculty of Science and Engineering, University of Groningen, 9712 CP Groningen, The Netherlands; 6 Division of Polar Ocean Sciences, Korea Polar Research Institute, 26 Songdomirae-ro, Yeonsu-gu, Incheon 21990, Korea

**Keywords:** Antarctic phytoplankton, Amundsen Sea Polynya, carbon flux, viral lysis, microzooplankton grazing, Southern Ocean

## Abstract

Whether phytoplankton mortality is caused by grazing or viral lysis has important implications for phytoplankton dynamics and biogeochemical cycling. The ecological relevance of viral lysis for Antarctic phytoplankton is still under-studied. The Amundsen Sea is highly productive in spring and summer, especially in the Amundsen Sea Polynya (ASP), and very sensitive to global warming-induced ice-melt. This study reports on the importance of the viral lysis, compared to grazing, of pico- and nanophytoplankton, using the modified dilution method (based on apparent growth rates) in combination with flow cytometry and size fractionation. Considerable viral lysis was shown for all phytoplankton populations, independent of sampling location and cell size. In contrast, the average grazing rate was 116% higher for the larger nanophytoplankton, and grazing was also higher in the ASP (0.45 d^−1^ vs. 0.30 d^−1^ outside). Despite average specific viral lysis rates being lower than grazing rates (0.17 d^−1^ vs. 0.29 d^−1^), the average amount of phytoplankton carbon lost was similar (0.6 µg C L^−1^ d^−1^ each). The viral lysis of the larger-sized phytoplankton populations (including diatoms) and the high lysis rates of the abundant *P. antarctica* contributed substantially to the carbon lost. Our results demonstrate that viral lysis is a principal loss factor to consider for Southern Ocean phytoplankton communities and ecosystem production.

## 1. Introduction

The Southern Ocean is a major sink for anthropogenic carbon dioxide (CO_2_), and summer phytoplankton blooms contribute to the efficiency of the biological pump [1,2,3]. Especially, the more abundant smaller-sized phytoplankton (<20 µm cell diameter, [4]) are responsible for a large portion of the primary production (up to 41–70%) [5,6], illustrating their ecological importance for carbon cycling. Productivity in the Southern Ocean varies regionally [7,8] with coastal polynyas (reoccurring open water regions surrounded by sea ice) in particular being areas of high productivity [9,10]. The Amundsen Sea Polynya (ASP) is one of the largest polynyas within the Amundsen Sea (between 100° and 135° W and south of 71° S [11]), generally opening up in the first half of November and closing again towards the end of March. The prymnesiophyte *Phaeocystis antarctica* often dominates the phytoplankton community in the ASP [12], as in most of the Amundsen Sea [13]. Diatoms are relatively more abundant in the sea ice zones [12,13] and in waters where *Phaeocystis* spp. do not bloom [14]. Other phytoplankton groups, i.e., cryptophytes, chlorophytes, and dinoflagellates, are considered less dominant taxonomic groups [15]. Note that smaller-sized phytoplankton within, for example, the chlorophytes (i.e., prasinophytes) may still be abundant in cell numbers [12].

The Southern Ocean and its regional seas are sensitive to global climate change-induced alterations, such as ocean temperature, turbulence, sea ice cover, and irradiance [16,17,18]. Currently, sea ice, ice shelves, and glaciers in the Amundsen Sea are rapidly melting [19,20,21], which is predicted to direct changes in ecosystem functioning and biogeochemical cycling [12,22,23]. Increased basal ice melt is foreseen in the Amundsen Sea, driven by the intrusion of the relatively warm (2 °C) circumpolar deep water (CDW) [24,25,26] due to enhanced wind-forcing [27]. Moreover, a trend of a poleward shift of westerly winds (which drive upwelling) was found [28], leading to the rapid warming of subsurface Antarctic coastal waters [29]. Sea ice has also been decreasing in the Amundsen Sea region, which is driven by a deepening of the Amundsen Sea Low, a climatological low-pressure area in the South Pacific Southern Ocean area (reviewed by [30]). It has been speculated that future primary productivity in Antarctic coastal seas will increase by enhanced iron input from glacier and sea-ice melt [31,32,33], which may favor *Phaeocystis antarctica* over diatoms [31]. Conversely, it has been argued that light rather than iron availability drives Amundsen Sea phytoplankton blooms, especially in the ASP [34,35]. For the West Antarctic region in general, a shift towards smaller-sized phytoplankton and an increase in diazotrophs and dinoflagellates have been predicted [36,37].

Antarctic phytoplankton (blooms) is, however, not only regulated by growth controlling factors (such as iron and light), but also by loss processes (grazing, sinking, and viral lysis). A study by Arteaga et al. [38] emphasized the importance of considering both division and loss rates when modeling future changes in Southern Ocean phytoplankton accumulation. Typically, grazing has been noted as the most important loss factor for Southern Ocean phytoplankton [22,39,40], with viral lysis rates at best considered small [41,42]. However, viral lysis was recently found to be an important loss factor of phytoplankton in the coastal waters of the Western Antarctic Peninsula [43]. In contrast to grazing, lytic viral infection shunts particulate organic matter away from higher trophic levels and instead stimulates the microbial food web by causing the release of the host cell content upon lysis [44,45]. This so-called viral shunt will result in reduced carbon export efficiency [46], although to what extent is currently not clear [47], as viral action may also result in aggregation and thereby stimulate carbon export [48,49,50,51]. Furthermore, the host-specific nature of phytoplankton viruses promotes species and strain succession and diversity, whereas microzooplankton grazers generally feed within prey size-ranges [52,53,54].

As phytoplankton forms the base of the food web, it is crucial to shed light on the ecological relevance of viruses for the highly productive but climate-sensitive Amundsen Sea. With this study, we aim to investigate the viral lysis rates of phytoplankton in the Amundsen Sea, both within and outside the ASP. We report on the share of viral lysis in relation to grazing for stations within and outside the ASP using the modified dilution assay, combined with flow cytometry and the size fractionation of phytoplankton populations. The results presented illuminate the importance of viral lysis and provide a better understanding of the Amundsen Sea phytoplankton carbon flow.

## 2. Materials and Methods

### 2.1. Study Area and Sampling

**Figure 1:**
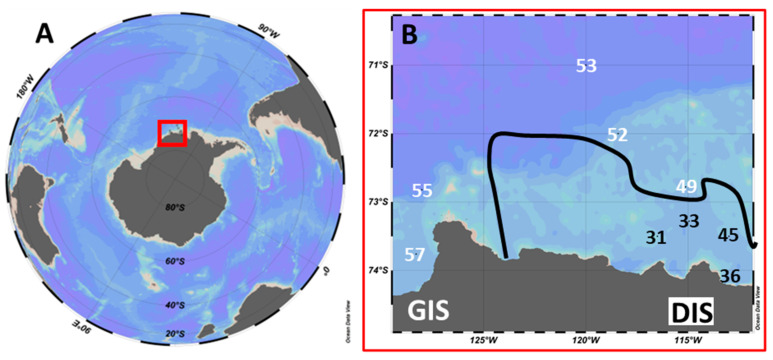
Location of the Amundsen Sea in the Southern Ocean (**A**) and a closeup of stations sampled within the Amundsen Sea (**B**). GIS = West Getz ice shelf region, DIS = Dotson ice shelf region. The black line shows the rough outline of the Amundsen Sea Polynya (ASP) during the period of sampling. Stations within the ASP are in black and stations outside the ASP are in white. Based on ice cover, density, and autofluorescence depth profiles, intermediate station 49 was treated as a non-ASP station (see Section 2).

The sampling of the water column was performed at specific stations (31, 33, 36, 45, 49, 52, 53, 55, and 57, from 18 January to 2 February 2018) while on board the South Korean Ice Breaker RV Araon during the ANA08B research expedition. Seawater was collected using the ‘Titan’ ultraclean (trace metal clean) sampling system with a Seabird CTD (SBE911+) [55], mounted with light-proof and trace-metal-clean polypropylene sampling bottles [56,57]. Subsamples (5–20 L, untreated) were obtained once the complete CTD sampling system was placed in the dedicated cleanroom environment (modified laboratory container). Sampling, temporary sample storage (in the dark and max 1 h), and sample processing were performed at 1 °C. Sampling depth was determined based on a non-trace-metal-clean CTD cast shortly before sampling, as the sensor data from the Titan CTD could only be downloaded once back on board (a cable was used without internal conductive wires and therefore an SBE 17 plus V2 Searam in a titanium housing provided power, saved the CTD data, and closed the sampling bottles at pre-programmed depths). For the stations outside the ASP (Figure 1), we aimed to sample below the reduced salinity surface layer (due to sea-ice melt) at the sub-surface Chlorophyll *a* (Chl *a*) autofluorescence maximum. The stations inside the ASP (Figure 1) were more deeply mixed and sampled in the mixed layer. All stations (average sampling depth 22 m (range: 11–36 m); Table 1) were sampled for phytoplankton community composition and specific growth and loss rates.

Stations 31–53 were located near the Dotson ice shelf (DIS), and stations 55 and 57 were near the West Getz ice shelf (GIS, Figure 1). Stations 31–45 lay within the ASP (based on ice coverage [58]). Based on cruise-average sea ice cover, station 49 was classified as an ASP station [58,59], however, date-specific ice coverage [60] implies a recent opening of sea ice (Figure S1). Taking into account the physical parameters, such as the density depth profile and the mixed layer depth (MLD, defined as a density difference of 0.05 kg per m^3^ compared to the density at 1 m depth), station 49 can be considered an intermediate station (Figure S1). The MLD of 23 m at station 49 was low compared to ASP stations (78.9 ± 26.2 m) and matched the MLDs of non-ASP stations (16.1 ± 6.1 m). Additionally, the Chl *a* autofluorescence depth profile for station 49 seems more comparable to non-ASP stations (Figure S1). Although we classified station 49 for the statistical analysis as a non-ASP station, none of our main conclusions were affected by whether station 49 was counted as an ASP or non-ASP station.

### 2.2. Nutrient Analysis

Dissolved inorganic macronutrient data were measured on-board, following Jeon et al. [59]. In short, dissolved inorganic phosphate and silicic acid concentrations were measured according to the Joint Global Ocean Flux Study (JGOFS) protocols [61], using a four-channel Auto-Analyzer (QuAAtro, Seal Analytical, Germany). Precisions for measurements were ±0.02 and ±0.28 µmol kg^−1^ for phosphate and silicic acid, respectively. Al-though nitrate and ammonium data were not available, the overall average concentrations of dissolved phosphate and silicate suggest non-limiting conditions [59], i.e., 1.7 ± 0.3 µM and 80 ± 8 µM, respectively (Table 1). Dissolved iron (dFe) was measured as described by van Manen et al. [62]. Samples were UV-digested for 4 h and pre-concentrated. Dissolved trace metal concentrations were calibrated using a mixed stock solution. Dissolved iron (dFe) concentrations were relatively low in surface waters for all stations (average 0.19 ± 0.09 nM; Table 1).

**Table 1:** Station location with physical (temperature, Temp.; and salinity, Sal.) and chemical (dissolved inorganic phosphate, PO4; silicate, Si(OH)_4_; and dissolved iron) characteristics at the sampling depth. Latitude and longitude are given in decimal degrees. Stations 36–45 were defined as ASP stations and stations 49–57 as non-ASP stations.

Station	Lat. (°S)	Long. (°W)	Depth (m)	Temp. (°C)	Sal. (PSS)	PO_4_ (µM)	Si(OH_4_) (µM)	dFe (nM)
31	73.5	116.5	15	−0.56	33.99	1.78	84.7	0.36
33	73.3	115.0	18	−0.32	33.95	1.57	78.1	0.09
36	74.2	113.3	28	−1.51	33.89	2.20	98.0	0.23
45	73.5	113.0	15	−0.69	33.85	1.51	77.7	0.19
49	72.8	115.1	21	−0.83	33.68	1.46	75.5	0.29
52	72.0	118.4	36	−1.58	33.89	2.07	78.5	0.11
53	71.0	120.0	30	−1.31	33.83	1.93	77.2	0.16
55	72.8	128.0	24	−1.54	33.50	1.29	70.3	0.08
57	73.8	128.3	11	−0.96	33.64	1.88	78.4	0.22

### 2.3. Phytoplankton Community Taxonomy

For phytoplankton pigment analysis, seawater samples were kept on ice until and during filtration. Whole water (untreated) and <20 µm (gently reverse-sieved) seawater samples were filtered through GF/F glass fiber filters (45 mm diameter; Whatman, Cytiva, Marlborough, MA, USA) using a vacuum pump (max 200 mbar). The average seawater volume needed to display color on the filter was 1.7 L (range: 0.75–2.7 L). Filters were double-wrapped in aluminum foil, snap-frozen in liquid nitrogen, and stored at −80 °C until analysis. Pigment analysis was performed via high-performance liquid chromatography (HPLC) with filters being freeze-dried and pigments subsequently dissolved in acetone [63]. A Zorbax Eclipse XDB-C8 column (3.5 μm particle size) was used for HPLC pigment separation [64] with detection based on retention time and diode array spectroscopy (Waters 996) at 436 nm. Pigments were identified based on retention time and the diode array spectroscopy of standards (DHI LAB products) and were subsequently quantified using the calibration curves of these standards. Phytoplankton taxonomic composition was determined using CHEMTAX (version 1.95; [65]), as described by Selz et al. [66]. For CHEMTAX analysis, the following pigments were used: Chlorophyll a, b, c2 and c3, Peridinin, 19′-Butanoyloxyfucoxanthin, Fucoxanthin, 19′-Hexanoylfucoxanthin, and Alloxanthin. The two groups of haptophyte pigment signatures used were pooled into one haptophyte group. For initial and final pigment ratios, see Table S6.

### 2.4. Phytoplankton Abundances

Samples for phytoplankton abundance were enumerated fresh using a 488 nm Argon laser Beckton–Dickinson FACSCalibur flow cytometer [67]. The trigger was set on red fluorescence and the different phytoplankton populations were discriminated based on plots of Chl *a* autofluorescence and side scatter using FCS express 5 (De Novo Software, Pasadena, CA, USA). Cryptophytes were differentiated by their orange phycoerythrin autofluorescence. In total, 17 different phytoplankton populations could be determined, 4 of which were cryptophytes.

Phytoplankton cell diameters were determined by size fractionation, i.e., a 20 mL sample was gently and sequentially filtered through 10, 8, 5, 3, 2, 1, 0.8, and 0.6 µm pore-size polycarbonate filters (Whatman, Cytiva, Marlborough, MA, USA) using a 25 mm syringe filter holder (Whatman, Cytiva, Marlborough, MA, USA) fitted in a disposable plastic syringe. Filtration was largely done by gravitation and only gentle pressure by the syringe stopper was applied when necessary (typically 0.8 and 0.6 µm filter). Each filtrate was subsampled for flow cytometric analysis. Cell counts were plotted against the filter pore size and the mean cell diameter was defined as the size shown by the median (50%) of cells retained for that population [68]. The 2 picophytoplankton populations (Phyto 1 and Phyto 2) displayed an average cell diameter of 1.0 and 2.0 µm, respectively. The remaining 15 nanophytoplankton populations (Phyto 3–16) ranged from 3.3 to 19.8 µm average cell diameter (Table 2). Phyto 4, 6, and 8 were cryptophytes with an average diameter of 3.3, 5.2, and 8.7 µm, respectively. Based on flow cytometric measurements using isolated *Phaeocystis antarctica* colonies on board, in combination with average cell size (Table 2 and [69,70]), we were able to discriminate Phyto 5 in the cytograms as *P. antarctica*-like (for ease referred to as *P. antarctica* in the rest of the paper). We acknowledge that *P. antarctica* is not the only *Phaeocystis* species present in Antarctic waters [71,72]; however, the colonies resembled *P. antarctica* and the Amundsen Sea is known for *P. antarctica* [31,73].

### 2.5. Loss Rates

Specific phytoplankton grazing and viral lysis rates were determined using the modified dilution assay in combination with flow cytometry [74,75]. In short, predation pressure by grazers and viruses is reduced by dilution and the apparent growth rates of the phytoplankton (obtained over 24 h) in the different dilutions are plotted against the dilution factor to acquire the specific loss rates (slope of the linear regression). Two types of filtrates were used to dilute the whole water, i.e., grazer-free filtrate was prepared by gravity filtration through a 0.45 µm cartridge (Sartorius, Goettingen, Germany), while grazer- and virus-free filtrate was prepared using a 30 kDa tangential flow cartridge (VivaFlow, Sartorius, Goettingen, Germany). The respective filtrates were gently mixed with 200 µm of sieved seawater to create a dilution series containing 20, 40, 70, and 100% of sieved seawater (in 1N HCl cleaned 1 L polycarbonate bottles, in triplicate). Subsamples (7 mL) for phytoplankton counting were then taken (T0), after which the bottles were topped up with the appropriate dilution and subsequently closed whilst preventing air bubble inclusions (using a convex silicon inlay). The dilution bottles were transported in the dark to an on-deck flow-through incubator, where they were attached to a slow-turning (0.5 rpm) wheel and incubated at ambient temperature and light conditions (neutral density screens were used to adapt the light level to the sampling depth). After 24 h, subsamples were taken again for phytoplankton enumeration (T24). The phytoplankton abundance samples were measured fresh using flow cytometry. The apparent growth rates were calculated from the T0 and T24 abundances and plotted against the actual dilution. The slope of the linear regression of the 0.45 µm series provides the specific grazing rate (G, d^−1^) and that of the 30 kDa series provides the loss rate due to grazing and viral lysis (G+V, d^−1^). The difference between both slopes provides the specific viral lysis rate (V, d^−1^). Additionally, gross growth rates (d^−1^) were determined by the *y*-axis intercept of the 30 kDa regression.

When the apparent growth rates of the phytoplankton of the 20% dilution series (triplicate bottles) were equal to or lower than the phytoplankton growth rates of the 40% dilution series, the 20% values were removed, as this indicates phytoplankton growth limitation of some kind. Similarly, when the phytoplankton growth rates of the 70% dilution series were equal to those of the 100% series, the latter were removed, as this indicates that predator (grazers, viruses) concentrations were not diluted enough to allow the increased apparent growth rates of the phytoplankton. To allow further analysis of the outcomes obtained from the modified dilution assay (e.g., averaging the rates), we considered (for each phytoplankton group in each experiment) only the successful results with rates for both grazing and viral lysis (including zero rates). Rates were obtained for 13 of the 16 phytoplankton populations (Phyto 1–7 and 9–14).

The numbers of phytoplankton cells produced and lost were calculated from the specific gross growth and loss rates, by using a simple model described by Biggs et al. [43]. In short, phytoplankton abundance dynamics during the 24-h incubation time are explained by the difference between the specific gross growth and total loss rates of phytoplankton in relation to phytoplankton abundance at the start of the incubation. The numbers of cells produced and lost were calculated by integrating the phytoplankton gross growth or total loss rates over time (d^−1^). The number of phytoplankton cells produced and lost (due to viral lysis and/or grazing) was then converted to organic carbon based on average cell diameter and using the conversion factors of 237 fg C µm^−3^ for picophytoplankton populations Phyto 1 and 2 and 196.5 fg C µm^−3^ for nanophytoplankton populations Phyto 3–17 [76,77].

### 2.6. Statistical Analyses

All statistical analyses were performed using R [78]. For phytoplankton total loss and microzooplankton grazing rates, statistical testing was performed using the linear models for the 30 kDa and 0.45 µm dilution series. A significant interaction means a significant difference between the linear models of the 30 kDa and 0.45 µm dilution series, i.e., a significant viral lysis rate of phytoplankton. For further analysis, both significant and non-significant rates were used. For this, rates were checked for normality (Shapiro–Wilk’s method) and homogeneity of variances (Fligner–Killeen’s test). Correlations were tested using linear regression (lin. reg.) models, whilst for comparison between phytoplankton groups within the dataset (e.g., different regions, different phytoplankton-size classes) the non-parametric Kruskal–Wallis test was used, which works with the median. The *n*-number for all statistical testing was at least 3 (when testing effects on specific phytoplankton populations) with 46 viral lysis and grazing rates of phytoplankton in the full dataset. Statistical results are reported in Table 3 (Kruskal–Wallis tests) and Table 4 (linear regressions).

## 3. Results and Discussion

### 3.1. Phytoplankton Community Composition

**Figure 2:**
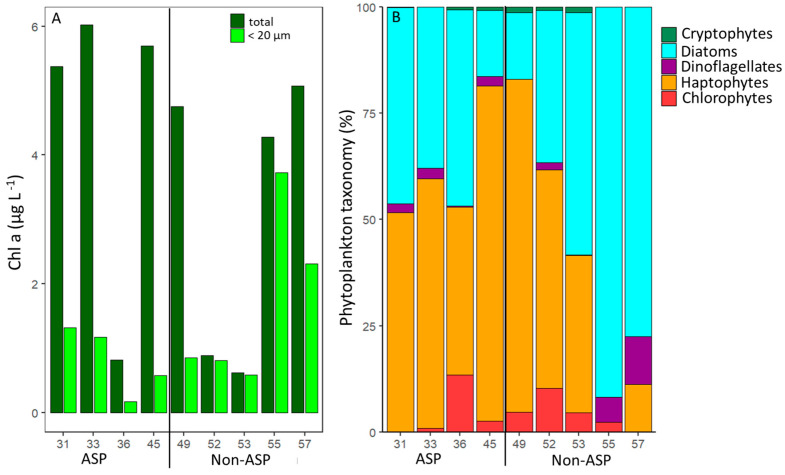
Chlorophyll *a* concentrations and taxonomic composition of the phytoplankton. (**A**) total and <20 µm Chl *a* concentrations. (**B**) Phytoplankton community composition for <20 µm size fraction. Stations 31–45 lie within the Amundsen Sea Polynya (ASP) and stations 49–57 outside of it.

Total and <20 µm Chl *a* concentrations ranged between 0.6 and 6.0 and 0.2 and 3.7 µg L^−1^, respectively (Figure 2). These Chl *a* concentrations compare well with published values reported for January (average of 7 µg Chl *a* L^−1^ [9]). A higher total Chl *a* concentration is typically reported for early January and December in the ASP [15,70,79]. Lee et al. [15] showed that the maximum Chl *a* value was around 13 µg Chl *a* L^−1^ in mid-January and decreased to 4 µg Chl *a* L^−1^ in February. The average total Chl *a* concentrations for stations 31–45 within the ASP were higher than for stations 49–57 located outside the ASP, i.e., 4.5 ± 2.10 and 3.1 ± 2.0 µg L^−1^, respectively (Table 3, test 1), which is supported by previous findings [80]. The higher amount of total Chl *a* within the ASP is likely due to higher dFe concentrations (mean 0.22 vs. 0.17 nM within and outside the ASP; Table 3, test 2) driven by the upwelling of relatively nutrient-rich CDW [32]. In contrast, the average Chl *a* concentration for <20 µm size fraction was higher outside the ASP (1.7 ± 1.2 µg Chl *a* L^−1^) compared to inside (0.8 ± 0.5 µg Chl *a* L^−1^; Table 3, test 3). This agrees with earlier observations that concentrations of smaller-sized phytoplankton (<5 µm) were lower within compared to outside of the ASP [6,81] and that larger phytoplankton cells were predominant at polynya stations [80,82]. The lower share of the <20 µm Chl *a* size fraction inside the ASP (10–47%, compared to 18–94% of total Chl *a* outside the ASP, *n* = 4 and 5, respectively; Table S7) is unlikely the result of reduced phytoplankton production. Instead, we hypothesize that it may reflect selective grazing for nutritious smaller-sized phytoplankton prey [10,81], which is supported by the higher grazing rates of phytoplankton within compared to outside of the ASP (Section 3.2; Table 3, test 4).

**Figure 3:**
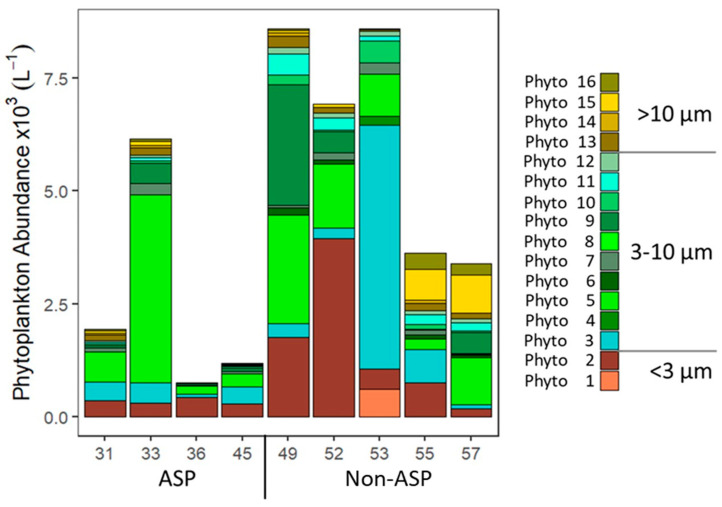
Phytoplankton populations, clustered in picophytoplankton (<3 um average cell diameter; Phyto 1 and 2), nano-sized phytoplankton populations 3-10 um (Phyto 3–13), and >10 um (Phyto 14–17). Stations 31–45 lie within the Amundsen Sea Polynya (ASP) and stations 49–57 outside of it.

Total phytoplankton abundance was highest at stations 49 and 53 (both outside the ASP, 8598 and 8595 cells mL^−1^, respectively; Figure 3) and lowest at station 36 (753 cells mL^−1^). In accordance with Chl *a* data for the <20 µm phytoplankton size fraction, total phytoplankton abundances (measured by flow cytometry; mostly <20 µm cell diameter) were significantly higher outside than within the ASP (mean: 6234 ± 2316 vs. 2508 ± 2166 cells mL^−1^; Table 3, test 5). Although Phyto 1, 4, and 8 (Figure 3) were only found outside of the ASP, their abundances (average of 67 ± 201, 22 ± 66, and 5 ± 11 cells mL^−1^, *n* = 1, 1, and 2, respectively) were not high enough to explain the difference in total phytoplankton abundances within and outside the ASP. No significant correlations were found between phytoplankton abundance and dissolved inorganic nutrients or salinity. The temperature was significantly higher inside compared to outside the ASP (mean: −0.8 ± 0.5 vs. −1.2 ± 0.3 °C; Table 3, test 6). Higher temperatures might lead to higher phytoplankton production [83,84]; however, the phytoplankton gross growth rates were not significantly different between ASP and non-ASP stations (Table S8). Furthermore, no significant correlation was observed between phytoplankton abundance and temperature, suggesting that the 0.4 °C difference in average temperature was not the (main) driver of phytoplankton abundance. The lower phytoplankton numbers within the ASP might thus be driven by the significantly higher total loss rates of the phytoplankton inside compared to outside the ASP (mean: 0.62 ± 0.27 d^−1^ versus 0.39 ± 0.27; Table 3, test 7 and see Section 3.2).

The phytoplankton community composition based on pigment taxonomy identified haptophytes and diatoms as the two most abundant taxa (Figure 2), in accordance with other studies in the Amundsen Sea [12,13,15,31]. However, the share of haptophytes in the <20 µm size fraction (compared to total Chl *a*) at stations 36, 45, 49, and 57 (Table S7) was lower, suggesting the presence of larger *P. antarctica* colonies at these stations (confirmed by the visual inspection of samples). The GIS region (stations 55 and 57) was dominated by diatoms (station 55 did not show haptophytes at all), whilst haptophytes were more abundant in the DIS region (stations 31–53). The stations inside the ASP displayed on average a higher share of haptophytes than the stations outside the APS (68 and 46% for total and 57 and 35 for <20 µm fraction only, respectively). *P. antarctica* is reported to have a high ability to adapt to changing light conditions [70], which likely explains why haptophytes were specifically dominating at the ASP stations (Figure 2 and Figure 3) with deeper MLD (78.9 ± 26.2 m in ASP vs. 16.1 ± 6.1 m in non-ASP stations). Flow cytometry abundance data confirmed the importance of *P. antarctica*., i.e., Phyto 5 displayed the highest average abundance in the ASP (1,265 ± 1300 cells mL^−1^). It was most abundant at stations 31, 33, and 57, with the highest share (68%) at station 33 (Figure 3). Station 33 lies in the center of the ASP, known for *P. antarctica* blooms [31,85,86], and within the impact zone of an outflow of relatively nutrient-rich circumpolar deep water from under the ice shelf [58,62]. Although dFe concentrations were not specifically elevated at this station (Table 1), it might be that the available dFe was rapidly assimilated by *P. antarctica* [31]. The relative importance of *P. antarctica* in the non-ASP stations 49 and 52 may be the result of the sampling date. Station 52 was at the time of sampling close to the ASP opening and station 49 was taken when the sea ice opened (29th of January) and thus on the edge of the ASP [58]. The MLD was not as deep (yet) as for the ASP stations (23 and 15 m for stations 49 and 52, respectively,) and increasing light availability due to the reduced sea ice cover can be expected to stimulate *Phaeocystis* colony formation [34,35]. Especially for Station 49, haptophytes also dominated the >20 µm size fraction (Table S7), confirming the presence of larger colonies. Although the melting sea ice has been described to provide micronutrients (such as iron and manganese, reviewed by [87]) which would stimulate Antarctic phytoplankton growth [31,32,33], van Manen et al. [62] reported that micronutrient supply by sea-ice melt during our sampling period was likely minimal.

Total phytoplankton abundance was highest in the non-ASP stations 49, 52, and 53, as a result of the high share of the smallest phytoplankton populations Phyto 1–3 (Figure 3). Phyto 1 was only observed at Station 53, which might be explained by its oceanic position [59,88]. Phyto 2 abundance was highest for station 52 (3956 cells mL^−1^; 57% of total) but the relative share was also high for stations 36 and 55 (57 and 21% of total abundances, respectively). Phyto 3 abundance was high only at station 53 (5408 cells mL^−1^ 63%), but at station 45 it was also relatively important, making up about one-third of the total abundance (33%). The small-sized Phyto 2 and Phyto 3 (Table 2) could represent chlorophytes, given that for these stations the share in chlorophytes was relatively large (Figure 2). Total chlorophyte concentrations ranged between 0 and 9% of total Chl *a* (Table S7), matching previous observations [13,15,89]. Of the other taxonomic groups, dinoflagellates showed a slightly higher share in the GIS region (station 55 and 57), and cryptophytes were only recorded in a low percentage within the DIS region (up to 2%, both within and outside of the ASP). The lower abundance of dinoflagellates in the DIS region compares well with previous observations [12]. The low share of the cryptophyte taxa (Figure 2) matched the overall low abundance of Phyto 4, 6, and 8, which were identified as cryptophytes by flow cytometry (Figure 3), as well as the literature reporting cryptophytes to be mainly found in the open ocean area of the Amundsen Sea [12]. Whilst Phyto 6 was found both within and outside the ASP, Phyto 4 and 8 were only found in non-ASP stations.

The flow cytometric phytoplankton community composition at stations 55 and 57 (GIS-region) differed from the other stations due to the high abundances of larger-sized Phyto 13–16 (average abundances for those stations being 139 ± 29, 38 ± 54, 765 ± 121, and 311 ± 83 cells mL^−1^, respectively). These Phyto groups (14.3–19.8 µm average diameter) showed comparable autofluorescence and side-scatter signatures (based on relative positions on flow cytometry scatter plots) as some Phyto groups identified as diatoms by Biggs et al. [90]. Therefore, we speculate them to be diatoms, which matches the 87 and 82% share of FCM-carbon and 92 and 77% share of diatom Chl *a* in the <20 um size fraction for stations 55 and 57, respectively (Tables S7 and S9).

Station 36 lies within the ASP but directly next to the ice shelf (Figure 1), where nutrient-rich modified CDW is diverted towards the surface and showed the lowest total phytoplankton abundances (753 cells mL^−1^). As CDW overflows onto the Amundsen Sea shelf, it is advected at depth from station 53 towards station 45. Modified CDW is upwelled and then flows from station 36 towards station 33 [58,62]. It might be that phytoplankton abundance is low at station 36 because the MLD is very deep at this station (113 m), potentially causing light limitation. The low phytoplankton abundance at station 36 is in agreement with the low Chl *a* concentration for the <20 µm fraction (Figure 2). The relatively low total Chl *a* concentration confirms previous studies reporting lower Chl *a* levels near the ice shelf compared to the mid-ASP region [15,34].

### 3.2. Phytoplankton Mortality Rates

Viral lysis and grazing rates were measured for 13 of the 16 phytoplankton populations, with all phytoplankton populations showing viral lysis (Table 5). The viral lysis rates were comparable to published rates for phytoplankton in the Antarctic and non-Antarctic seas [42,43,75,91,92,93,94]. As with all methods, the modified dilution assay also has some constraints [74,95,96]. The assay is based on new viral infections and the cell lysis of the infected hosts within 24 h [74,91]. This restriction may lead to the overestimation of the viral lysis rates in the case of reinfection and host cell lysis within 12 h; however, known eukaryotic phytoplankton host-virus model systems indicate that this rarely occurs [97]. Alternatively, the underestimation of the measured viral lysis rates is possible when (the majority) of the viral-induced mortality of the phytoplankton occurs after 24 h. For example, current diatoms host-virus model systems in culture indicate long latent periods [98,99] with the consequently relatively late lysis of the host cells. However, we found substantial viral lysis rates for Phyto 12–14, identified as potential diatoms, which implies that not all diatom viruses have long latent periods (and result in late host lysis).

Often, viral lysis and grazing losses of the phytoplankton occurred simultaneously, except for *P. antarctica* (Phyto 5), for which losses seemed driven by either viral lysis or grazing (depending on the station). This supports the findings by Biggs et al. [43] that the high viral lysis of *Phaeocystis* spp. in Ryder Bay (Western Atlantic Peninsula) coincided with low rates of grazing and vice versa. Potential explanations include preferential grazing on, or avoidance of, infected *P. antarctica* cells [100,101,102]. Alternatively, colony formation may have reduced the chance of infection, even at a low colonial cell number, as shown for *P.s globosa* [103,104]. If this principle also holds for other *Phaeocystis* species, even minor variations in the number of small-sized *P. antarctica* colonies may affect the share of viral lysis for Phyto 5. It might also be that the microzooplankton grazers feeding on *P. antarctica* are in turn preyed upon by larger-sized zooplankton [86,105]. The pattern of one of the loss factors (viral lysis or grazing) dominating may not be restricted to *P. antarctica*, i.e., Phyto 2 displayed either lysis or grazing prevalence six out of seven times (Table 5).

**Figure 4:**
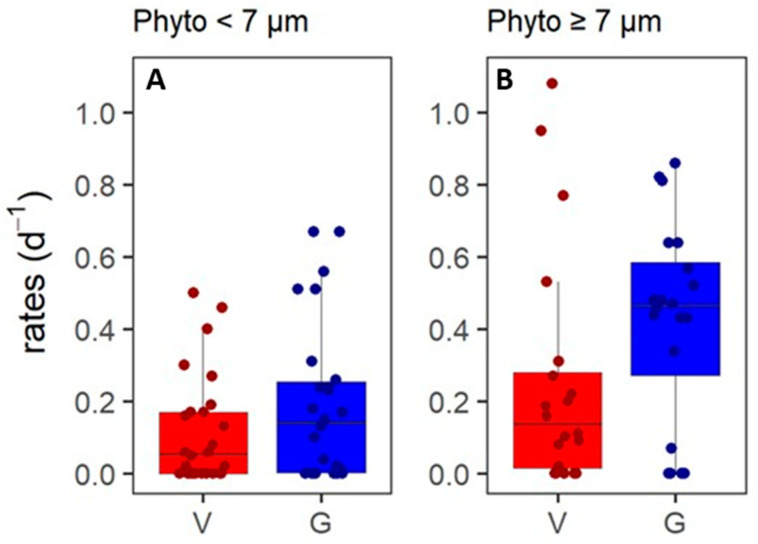
Viral lysis (V) and microzooplankton grazing (G) of phytoplankton for different size ranges. (**A**) Phytoplankton < 7 µm (*n* = 26). (**B**) Phytoplankton ≥ 7 µm (*n* = 20). The black line shows the median, the boxes show the interquartile range (IQR), and the whiskers show minimum and maximum values without outliers. Outliers are defined as outside 1.5 × IQR. The colored dots show the data points.

The grazing rates of the phytoplankton populations displayed a weak positive correlation to phytoplankton cell size (Figure S2; Table 4, test 1), with high rates, especially, for the larger phytoplankton (Table 5). This suggests selective grazing on this larger phytoplankton prey size class and/or additional predation pressure by larger zooplankton [86]. Grazers feed within specific size ranges [52,54], whilst viral lysis is host-specific [106]. Indeed, no significant correlation between viral lysis rates and phytoplankton cell size was found. Based on Figure S2, we grouped the Phyto populations into two size classes: < 7 µm average cell diameter and ≥7 µm cell size (*n* = 26 and 20, Figure 4). We found significantly higher grazing rates for the ≥7 µm phytoplankton (Phyto 7–16), as compared to the <7 µm Phyto groups (0.42 ± 0.28 and 0.20 ± 0.21 d^−1^, respectively; Table 3, test 11). This difference seems to be driven by higher grazing rates of the larger-sized Phyto 7–14 within the ASP (mean: 0.59 ± 0.21 vs. 0.26 ± 0.19; Table 3, test 12). Within the ≥7 µm-size class, only Phyto 7, 9, and 10 showed significantly higher grazing than lysis rates within the population (Table 3, test 8–10).

**Figure 5:**
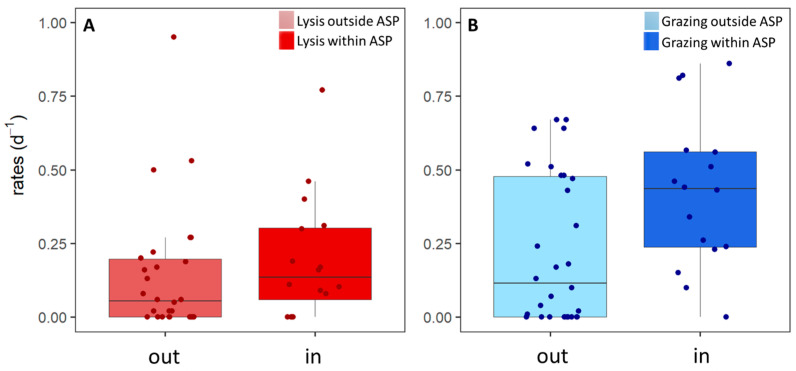
Viral lysis (**A**) and microzooplankton grazing (**B**) rates from stations outside and within the Amundsen Sea Polynya (ASP). The black line shows the median, the boxes show the interquartile range (IQR), and the whiskers show minimum and maximum values without outliers. Outliers are defined as outside 1.5 × IQR. The colored dots show the direct data points. *n*-numbers were 30 and 16 for outside and within the ASP, respectively.

Total phytoplankton losses were significantly higher inside compared to outside the ASP (0.62 vs. 0.39 d^−1^, respectively; Table 3, test 4), which was mainly driven by grazing (mean 0.42 ± 0.26 d^−1^ inside versus 0.23 ± 0.25 d^−1^ outside ASP; Figure 5 and Table 3, test 7). The lower average total phytoplankton abundance in the ASP (2508 ± 2166 and 6234 ± 2316 cells mL^−1^ in and out ASP, respectively; Table 3, test 5) were likely driven by these higher grazing rates. Grazing rates were (weakly) correlated to water temperature (Figure S3; Table 4, test 2), matching the model-based observations for eutrophic waters by Chen et al. [107]. Phytoplankton gross growth rates were not significantly different in and outside of the ASP (mean: 0.30 ± 0.38 and 0.45 ± 0.43 d^−1^, respectively, Table S8). Therefore, we hypothesize that the difference in the grazing of the phytoplankton in and outside the ASP could be related to increased metabolic rates of the zooplankton predators at higher temperatures in the ASP (average temperature −1.2 vs. −0.8 °C; Table 3, test 6) [108]. Additionally, the growth of grazers has been shown to decrease faster with decreasing temperature than phytoplankton production [109]. As we did not count zooplankton, we cannot exclude the possibility of higher zooplankton abundances as an additional reason for the increased grazing rates of the phytoplankton within the ASP. No significant differences were found in the total viral lysis rates of phytoplankton within versus outside of the ASP (Figure 5). It might be that viral lysis is closely coupled to phytoplankton host gross growth, as virus proliferation is, after all, dependent on host metabolism [110].

**Figure 6:**
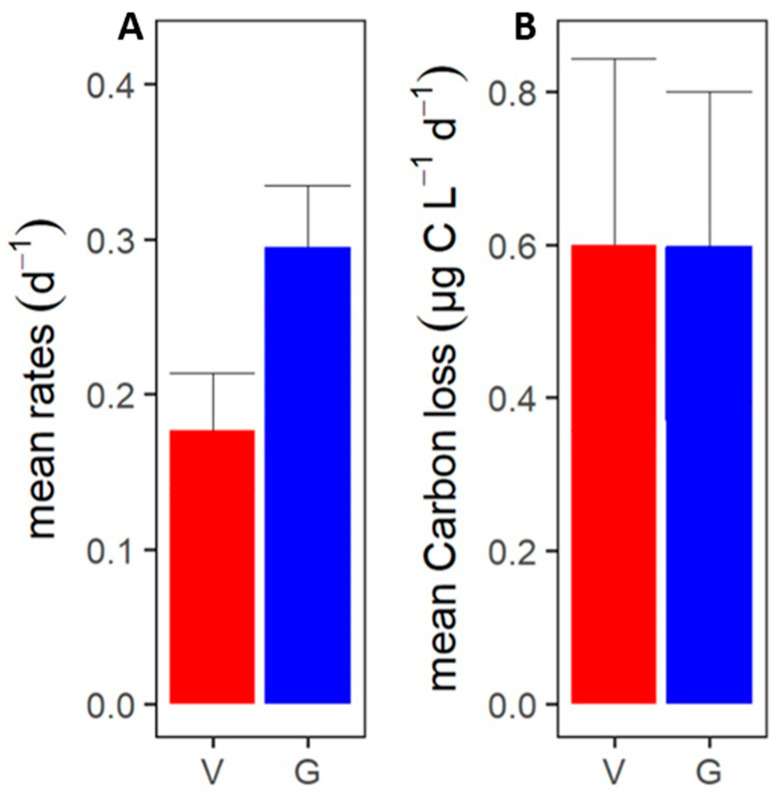
Mortality rates for all phytoplankton populations and all stations. (**A**) Mean specific viral lysis (V) and microzooplankton grazing (G) rates. (**B**) Mean viral lysis and grazing mediated phytoplankton carbon losses. The error bar shows the standard deviation (only positive SD is shown). The *n*-number for both specific viral lysis and grazing rates as well as carbon loss was 46.

On the whole, the total losses of phytoplankton correlated positively with the phytoplankton gross growth rates (Figure S4; Table 3, test 3). Loss rates were on average higher than the phytoplankton gross growth rate (0.46 vs. 0.35 d^−1^, respectively; Table S8). Loss rates were consequently negatively correlated to phytoplankton abundances (Figure S5; Table 3, test 4), whilst gross growth rates were not. This implies a resultant decline in phytoplankton cell standing stock, which relates well with published observations that phytoplankton blooms in the ASP are generally in decline in mid-January [15,22,89,111]. Grazing contributed most to the average total loss rate of the phytoplankton (63%) and the grazing rates were comparable to published microzooplankton grazing in this region (average 0.29 vs. 0.24 d^−1^, [22]). The average viral lysis rate (0.18 ± 0.25 d^−1^, Figure 6A) was lower than the average grazing rate (Table 3, test 13). Compared to an earlier study by Evans et al. [42], the phytoplankton loss rates in our study are actually higher, which may be the result of regional differences and distance to the shore (more open waters in the Southern Ocean versus the coastal Amundsen Sea). The phytoplankton grazing and viral lysis rates in our study are, however, largely comparable to the reported rates for phytoplankton in the coastal waters of the Western Antarctic Peninsula from mid-January to early February [43]. Our study fortifies the importance of viral lysis as a loss factor for Antarctic coastal marine phytoplankton.

### 3.3. Phytoplankton Carbon Loss

Even though the average specific phytoplankton lysis rate was smaller than the average grazing rate (Figure 6; Table 3, test 13), viral lysis was an equally important loss factor of the phytoplankton as grazing when expressed in cellular carbon, i.e., an average of 0.6 µg C L^−1^ d^−1^ for both (± 1.4 and ± 1.7 µg C L^−1^ d^−1^ for viral lysis and grazing, respectively; Figure 6). This is largely due to several high lysis rates in combination with high abundances of *P. antarctica* (Phyto 5) and the larger-sized Phyto 11. It should be noted, however, that even though the average rate was similar, the median amount of phytoplankton carbon grazed tested significantly higher than the amount of phytoplankton carbon lysed (0.20 vs. 0.07 µg C L^−1^ d^−1^; Table 3, test 14). Still, our results exemplify that a few relatively large viral lysis events can considerably impact the carbon flow. This illustrates that it is not only important to consider the actual rates but also phytoplankton host abundance and cell size to better understand the ecological impact of viral lysis in these productive Antarctic coastal waters. Ideally, one would also perform temporal studies to obtain insight into how frequent these high lysis events are.

The high share of viral lysis in total phytoplankton carbon loss indicates that it is an important loss factor of phytoplankton to be considered for carbon budgeting and modeling in the Southern Ocean. Large amounts of organic carbon are shunted towards the microbial loop instead of being transferred to higher trophic levels this study, [42,43]. The viral lysis of phytoplankton may thus partially explain the reported low carbon sequestration within the ASP region [112]. Moreover, the high host specificity of viruses [53,54] shapes phytoplankton community composition, which makes it an important factor to consider for projections of how phytoplankton community compositions will shift in response to global climate change [90,113]. As the fate of organic carbon is very different when phytoplankton is lysed compared to grazed [44,45], and the knowledge of the ecological impact of viral lysis for Antarctic phytoplankton is still limited, it is highly important for future predictions of carbon cycling to further investigate the importance of phytoplankton viruses in different sections of the Southern Ocean as well as during different seasons and years. Besides, most of the marine viruses infect the numerically dominant bacteria and it has been shown for Antarctic marine systems that bacterial viral lysis rates were on average higher than the grazing rates of bacteria [114]. In combination, the viral lysis of phytoplankton and bacteria might be more important than grazing when considering carbon losses. We recommend future studies to include viral lysis and grazing rate measurements of bacteria (and other prokaryotes).

Viral lysis may not only stimulate the microbial food web, but it may also lead to increased carbon export due to the formation of sticky aggregates [48,115]. This so-called viral shuttle was shown in laboratory experiments for diatoms [51]. For diatoms in iron-limited waters (such as the Southern Ocean), another shuttle system (the so-called viral shuttle) has also recently been suggested, based on a combination of increased silicate in diatom shells and delayed virus-induced mortality [116,117]. Both the viral shunt and viral shuttle, however, would still decrease the trophic transfer efficiency compared to grazing-dominated systems. As the ASP has high densities of krill [118], considered the ‘keystone species’ in the Southern Ocean, a viral-mediated decrease in trophic transfer efficiency might have severe implications on the food web, up to large animals such as penguins and whales.

## Figures and Tables

**Table 2:** Average diameters of phytoplankton populations. Based on their average cell diameter, Phyto 1 and 2 were defined as picophytoplankton and Phyto 3–17 as nanophytoplankton. Phyto 4, 6, and 8 were defined as cryptophytes, based on their orange autofluorescence.

Phyto	Average Diameter (µm)
1	1.0
2	2.0
3	3.3
4	3.3
5	4.2
6	5.2
7	8.6
8	8.7
9	9.1
10	9.6
11	10.0
12	10.0
13	14.3
14	15.4
15	19.0
16	19.8

**Table 3:** Statistical data from Kruskal–Wallis testing. T = test number. Dep = dependent variables: Chl *a* = Chlorophyll *a* (µg mL^−1^); dFe = dissolved iron (nM); Chl *a* < 20 µm = Chlorophyll *a* (µg L^−1^); G = grazing rate (d^−1^); Abun = phytoplankton cell abundance (mL^−1^); Temp = water temperature (°C) at sampling depth; TL = total loss rate (d^−1^); P7, P9, P10 = Phyto population 7, 9, and 10; C = phytoplankton carbon (µg L^−1^); ASP = Amundsen Sea Polynya. Expl = explanatory variables: in = ASP stations; out = non-ASP stations; G or V = specific grazing rate (d^−1^) or viral lysis rate (d^−1^); G = grazing rate (d^−1^); V = viral lysis rate (d^−1^); size = phytoplankton cell size; <7 = phytoplankton < 7 µm cell diameter; ≥7 = phytoplankton ≥ 7 µm cell diameter. H = Kruskal–Wallis test statistic; df = degrees of freedom; *p* = *p*-value of significance; SD = standard deviation; *n* = number of samples.

T	Dep	Expl	H	df	*p*	Mean ± SD		Median		*n*	
						in	out	in	out	in	out
1	Chl *a*	ASP	45.9	1	1.2 × 10^−11^	4.5 ± 2.1	3.1 ± 2.0	5.5	4.3	4	5
2	dFe	ASP	23.0	1	1.6 × 10^−6^	0.22 ± 0.10	0.17 ± 0.08	0.21	0.16	4	5
3	Chl a < 20 µm	ASP	20.4	1	6.3 × 10^−6^	0.8 ± 0.5	1.7 ± 1.2	0.9	0.9	4	5
4	G	ASP	5.4	1	0.02	0.42 ± 0.26	0.23 ± 0.25	0.44	0.13	16	30
5	Abun	ASP	81.6	1	2.2 × 10^−16^	2.5 ± 2.2	6.2 ± 2.3	1.6	6.9	4	5
6	Temp	ASP	62.5	1	2.7 × 10^−15^	−0.8 ± 0.5	−1.2 ± 0.3	−0.6	−1.3	4	5
7	TL	ASP	13.0	1	0.0003	0.62 ± 0.27	0.39 ± 0.27	0.57	0.37	16	30
**T**	**Dep**	**Expl**	**H**	**df**	***p***	**G**	**V**	**G**	**V**	**G**	**V**
8	TL P7	G or V	6.0	1	0.01	0.47 ± 0.25	0.09 ± 0.09	0.47	0.08	7	7
9	TL P9	G or V	3.9	1	0.05	0.71 ± 0.22	0.16 ± 0.16	0.82	0.16	3	3
10	TL P10	G or V	3.9	1	0.05	0.53 ± 0.10	0.13 ± 0.13	0.52	0.09	3	3
**T**	**Dep**	**Expl**	**H**	**df**	***p***	**<7**	**≥7**	**<7**	**≥7**	**<7**	**≥7**
11	G	size	5.5	1	0.01	0.20 ± 0.21	0.42 ± 0.28	0.24	0.47	26	20
12	G ASP	size	0.8	1	0.01	0.26 ± 0.19	0.59 ± 0.21	0.23	0.51	8	8
**T**	**Dep**	**Expl**	**H**	**df**	***p***	**G**	**V**	**G**	**V**	**G**	**V**
13	TL	G or V	4.7	1	0.03	0.29 ± 0.27	0.18 ± 0.25	0.24	0.19	46	46
14	TL C	G or V	3.6	1	0.06	0.60 ± 1.36	0.61 ± 1.67	0.2	0.07	46	46

**Table 4:** Statistical data from linear models. T = test number. Dep = dependent variables: G = grazing rate (d^−1^); TL = total loss rate (d^−1^). Expl = explanatory variables: Size = average cell diameter (µm); Temp = temperature (°C); Gross growth = gross growth rate (d^−1^); Abun = phytoplankton cell abundance (mL^−1^). Slope = slope of regression line; r^2^ = statistical measure of fit; *p* = *p*-value of significance; *n* = number of samples.

T	Dep	Expl	slope	r^2^	*p*	*n*
1	G	Size	0.03	0.12	0.01	46
2	G	Temp	0.26	0.18	0.004	46
3	TL	Gross growth	0.41	0.21	0.001	46
4	TL	Abun	−9.4 × 10^−5^	0.09	0.04	46

**Table 5:** Specific viral lysis and microzooplankton grazing rates (d^−1^) for the different phytoplankton populations in the Amundsen Sea. Stations 31–45 were located within the ASP, stations 55 and 57 near the GIS, and the other stations are located at the DIS.

Station	Gate	Lysis (d^−1^)	Grazing (d^−1^)	Station	Gate	Lysis (d^−1^)	Grazing (d^−1^)
31	Phyto 2	0.08	0.51	52	Phyto 2	0	0.13
	Phyto 3	0.19	0.26		Phyto 3	0	0
	Phyto 5	0	0.23		Phyto 5	0	0
	Phyto 7	0	0.81		Phyto 7	0	0.48
	Phyto 9	0.31	0.46		Phyto 11	0	0
33	Phyto 2	0.4	0.56		Phyto 13	0.19	0
	Phyto 3	0.17	0.15	53	Phyto 1	0.27	0.02
	Phyto 5	0.3	0		Phyto 2	0.17	0
	Phyto 9	0	0.82		Phyto 3	0	0.18
36	Phyto 2	0.46	0.1		Phyto 4	0.02	0.04
	Phyto 9	0.16	0.86		Phyto 5	0.02	0.01
45	Phyto 6	0	0.24		Phyto 7	0	0
	Phyto 7	0.11	0.44	55	Phyto 2	0	0.24
	Phyto 10	0.09	0.43		Phyto 3	0.06	0
	Phyto 11	0.77	0.34		Phyto 5	0	0.51
	Phyto 14	0.1	0.57		Phyto 6	0.05	0
49	Phyto 2	0	0.17		Phyto 7	0.08	0.43
	Phyto 3	0.16	0.31		Phyto 11	0.53	0
	Phyto 5	0.5	0	57	Phyto 5	0	0.1
	Phyto 6	0.13	0.67		Phyto 6	0.06	0.67
	Phyto 7	0.22	0.64		Phyto 7	0.2	0.47
	Phyto 10	0.02	0.64		Phyto 10	0.27	0.52
	Phyto 11	1.08	0.48		Phyto 12	0.95	0.07

## Data Availability

The data presented in this study are available on request from the corresponding author.

## Supplementary Materials

The following are available online at https://www.mdpi.com/article/10.3390/microorganisms10101967/s1, Figure S1: Characteristics of station 49; Figure S2: Correlation between specific grazing rates of the different phytoplankton populations and the average phytoplankton cell diameter (size) of the particular Phyto populations; Figure S3: Correlation between the specific grazing rates of the different phytoplankton populations and temperature at location and depth of sampling; Figure S4: Correlation between specific total loss and gross growth rates of the different phytoplankton populations; Figure S5: Correlation of specific total loss rates of the different phytoplankton populations and the total phytoplankton abundances; Table S6: Initial and final pigment ratios relative to Chl *a*; Table S7: Taxonomic phytoplankton community composition (% Chl *a*) for total phytoplankton and <20 µm size fraction; Table S8: Specific viral lysis, microzooplankton grazing and gross growth rates (d^−1^) for the different phytoplankton populations in the Amundsen Sea; Table S9: Relative carbon contribution (%), determined from flow cytometry counts and size fractionation, for Phyto 1-16 at each sampling station.
