# Supplementary material for: Ecological Importance of Viral Lysis as a Loss Factor of Phytoplankton in the Amundsen Sea
Source: Microorganisms. 2022 Oct 5;10(10):1967. doi: 10.3390/microorganisms10101967 (PMC9608467; doi:10.3390/microorganisms10101967)
Supplement: Supplementary file 1 [file microorganisms-10-01967-s001.zip › microorganisms-1868940-supplementary.pdf]

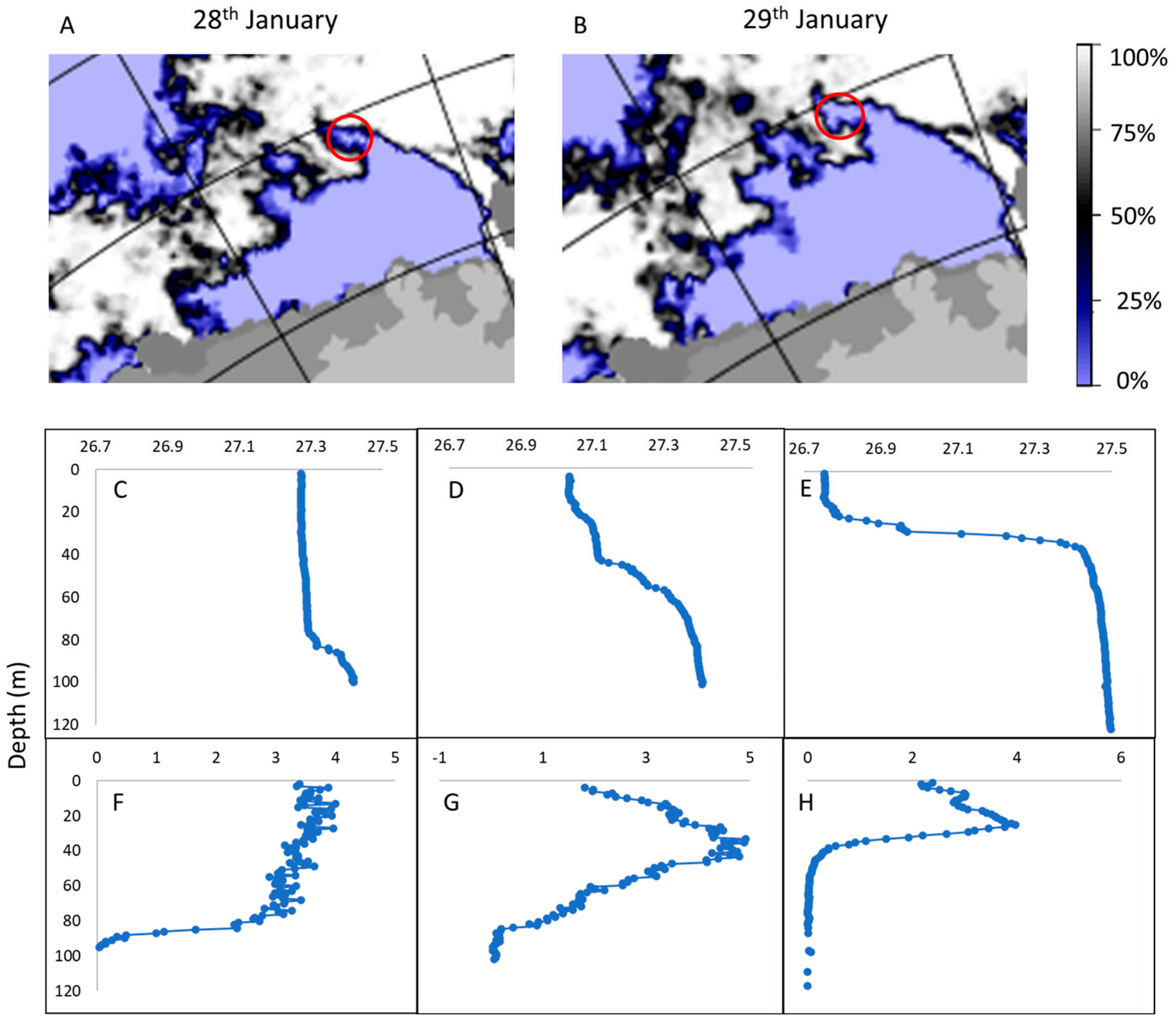

Figure S1. Characteristics of station 49. A & B: Sea ice coverage for station 49 (red circle) the day before (28th) and the day of sampling (29th of January). Data was derived from AMSR2 using the ARTIST sea ice algorithm; grid size 6.25 km (Spreen et al. [52]). C – H: Depth profile of density (C – E) and Chl a autofluorescence (F – H) for a typical ASP-station (C and F), for station 49 (D and G) and for a typical non-ASP station (E and H).

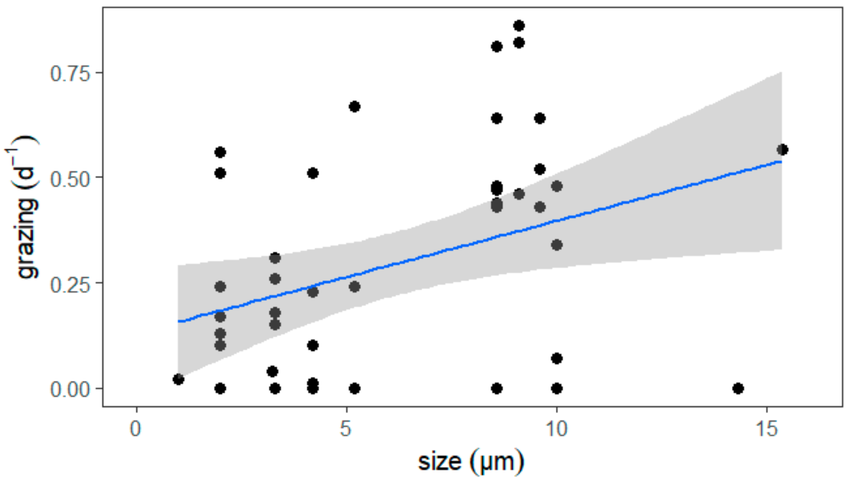

Figure S2. Correlation between specific grazing rates of the different phytoplankton populations and the average phytoplankton cell diameter (size) of the particular Phyto populations. $p = 0.02$, slope = 0.03, $r^2 = 0.12$. The blue line shows the regression slope, the grey area shows the 95% confidence interval.

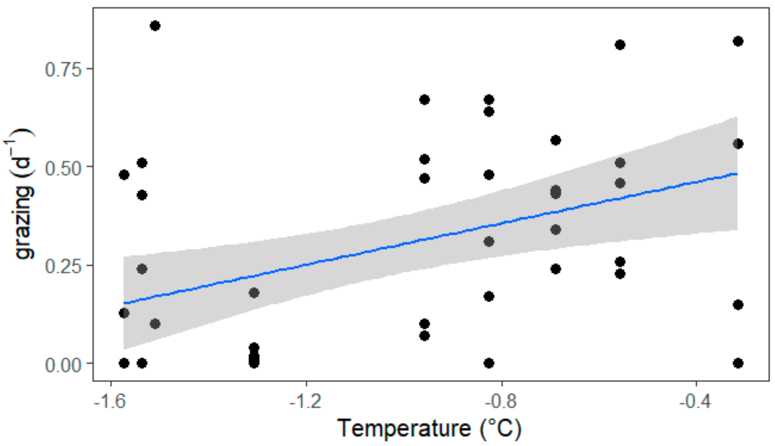

Figure S3. Correlation between specific grazing rates of the different phytoplankton populations and temperature at location and depth of sampling. $p = 0.004$, slope = 0.26, $r^2 = 0.18$ The blue line shows the regression slope, the grey area shows the 95% confidence interval.

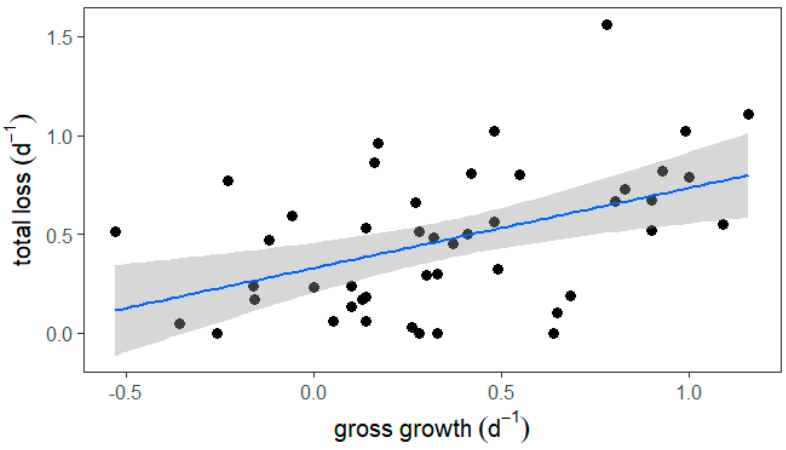

Figure S4. Correlation between specific total loss and gross growth rates of the different phytoplankton populations. p = 0.001, slope = 0.41, r^2 = 0.21. The blue line shows the regression slope, the grey area shows the 95% confidence interval.

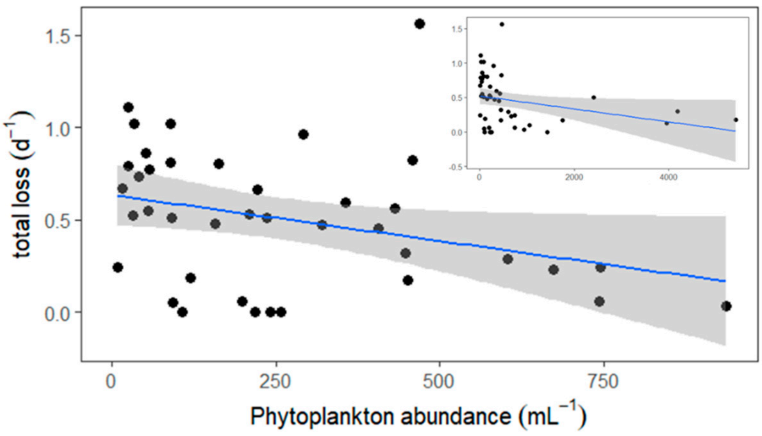

Figure S5. Correlation of specific total loss rates of the different phytoplankton populations and the total phytoplankton abundances. $p = 0.04$, slope = -9.4×10^{-5}, $r^2 = 0.1$. When taking out abundances over 1000 cells mL^{-1}, the correlation was still significant ($p = 0.03$, slope = -0.0005, $r^2 = 0.09$). The inlay shows the regression line when high abundant phytoplankton are included. The blue line shows the regression slope, the grey area shows the 95% confidence interval.

Table S6. Initial and final pigment ratios relative to Chl *a*: Perid = Peridinin, 19butfu = 19' – Butanoyloxyfucoxanthin, Fucox = Fucoxanthin, 19hexfu = 19' – Hexanoylfucoxanthin, Allox = Alloxanthin, Chl_c3 = Chlorophyll *c3*, Chl_c2 = Chlorophyll *c2*, Chl_b = Chlorophyll *b*.

	Phyto group	Perid	19butfu	Fucox	19hexfu	Allox	Chl_c3	Chl_c2	Chl_b
Initial	Chlorophytes	0.00	0.00	0.00	0.00	0.00	0.00	0.00	0.71
	Dinoflagellates	0.69	0.00	0.00	0.00	0.00	0.00	0.18	0.00
	Cryptophytes	0.00	0.00	0.00	0.00	0.29	0.00	0.14	0.00
	Haptophytes_1	0.00	0.01	0.30	0.65	0.00	0.14	0.13	0.00
	Haptophytes_2	0.00	0.30	0.30	0.10	0.00	0.12	0.13	0.00
	Diatoms	0.00	0.00	0.50	0.00	0.00	0.00	0.07	0.00
Final	Chlorophytes	0.00	0.00	0.00	0.00	0.00	0.00	0.00	0.71
	Dinoflagellates	0.69	0.00	0.00	0.00	0.00	0.00	0.18	0.00
	Cryptophytes	0.00	0.00	0.00	0.00	0.29	0.00	0.14	0.00
	Haptophytes_1	0.00	0.01	0.27	0.96	0.00	0.26	0.29	0.00
	Haptophytes_2	0.00	0.30	0.14	0.00	0.00	0.12	0.13	0.00
	Diatoms	0.00	0.00	0.69	0.00	0.00	0.00	0.20	0.00

Table S7. Taxonomic phytoplankton community composition (% Chl *a*) for total phytoplankton and < 20 μm size fraction.

Fraction	Group	Stations								
		**31**	**33**	**36**	**45**	**49**	**52**	**53**	**55**	**57**
Total	Chlorophytes	0	0	3	0	0	9	4	2	0
	Dinoflagellates	0	4	0	0	0	0	0	7	7
	Cryptophytes	1	1	1	0	1	1	2	0	1
	Diatoms	53	30	37	0	0	40	51	91	54
	Haptophytes	46	65	59	100	99	50	44	0	38
< 20 μm fraction	Chlorophytes	0	1	14	3	5	10	5	2	0
	Dinoflagellates	2	2	0	2	0	2	0	6	11
	Cryptophytes	0	0	1	1	1	1	1	0	0
	Diatoms	46	38	46	15	16	36	57	92	77
	Haptophytes	52	59	39	79	78	51	37	0	11

Table S8. Specific viral lysis, microzooplankton grazing and gross growth rates (d^{-1}) for the different phytoplankton populations in the Amundsen Sea. No data means no rates were obtained as population was not present or at very low numbers, or the assay failed. Note Phyto 8, 15 and 16 are not listed in the table as we have no loss rates data for these phytoplankton populations. Asterisks show when rates were statistically significant from 0 (grazing and gross growth) or from the grazing regression (viral lysis) (* = p<0.1, ** = p<0.05).

**Phyto**	**1**				**2**				**3**			
**Station**	**L**	**G**	**GG**	**TL**	**L**	**G**	**GG**	**TL**	**L**	**G**	**GG**	**TL**
31					0.08	0.51	-0.06**	0.59**	0.19	0.26	0.37**	0.45**
33					0.4	0.56	0.17*	0.96*	0.17	0.15	0.49	0.32
36					0.46	0.1	0.48**	0.56**				
45												
49					0	0.17*	0.13	0.17	0.16	0.31**	-0.12	0.47
52					0	0.13	0.10**	0.13**	0	0	-0.26	0
53	0.27*	0.02	0.30**	0.29**	0.17	0	-0.16	0.17	0	0.18**	0.14	0.18
55					0	0.24	0.10**	0.24**	0.06	0	0.05	0.06
57												

**Phyto**	**4**				**5**				**6**			
**Station**	**L**	**G**	**GG**	**TL**	**L**	**G**	**GG**	**TL**	**L**	**G**	**GG**	**TL**
31					0	0.23	0	0.23				
33					0.30*	0	0.33**	0.30**				
36												
45									0	0.24	-0.16	0.24
49					0.5	0	0.41	0.5	0.13	0.67**	0.55**	0.80**
52					0	0	0.33	0				
53	0.02	0.04	0.14	0.06	0.02	0.01	0.26	0.03				
55					0	0.51**	0.28**	0.51**	0.05	0	-0.36	0.05
57					0	0.1	0.65	0.1	0.06	0.67*	0.83*	0.73*

**Phyto**	**7**				**9**				**10**			
**Station**	**L**	**G**	**GG**	**TL**	**L**	**G**	**GG**	**TL**	**L**	**G**	**GG**	**TL**
31	0	0.81*	0.42**	0.81**	0.31	0.46	-0.23*	0.77*				
33					0	0.82**	0.93*	0.82*				
36					0.16	0.86	0.48*	1.02				
45	0.11	0.44	1.09	0.55					0.09	0.43*	0.9	0.52
49	0.22	0.64*	0.16	0.86					0.02	0.64**	0.27**	0.66**
52	0	0.48*	0.32*	0.48*								
53	0	0	0.64	0								
55	0.08	0.43	-0.53	0.51								
57	0.2	0.47	0.9	0.67					0.27	0.52	1	0.79

Phyto	11				12				13			
Station	L	G	GG	TL	L	G	GG	TL	L	G	GG	TL
31												
33												
36												
45	0.77**	0.34**	1.16**	1.11**								
49	1.08**	0.48**	0.78**	1.56**								
52	0	0	0.28	0					0.19	0	0.68	0.19
53												
55	0.53	0	0.14**	0.53**								
57					0.95	0.07	0.99*	1.02*				

Phyto	14			
Station	L	G	GG	Loss
31				
33				
36				
45	0.1	0.57	0.8	0.67
49				
52				
53				
55				
57				

Table S9. Relative carbon contribution (%), determined from flow cytometry counts and size fractionation, for Phyto 1-16 at each sampling station.

Phyto	31	33	36	45	49	52	53	55	57
1	0	0	0	0	0	0	0	0	0
2	1	0	12	3	1	7	1	1	0
3	4	2	6	12	1	1	34	1	0
4	0	0	0	0	0	0	1	0	0
5	11	29	26	14	9	14	10	1	4
6	0	0	0	1	1	1	0	1	0
7	6	7	5	11	8	6	10	1	0
8	0	0	0	0	0	0	0	0	1
9	4	15	23	14	47	21	0	1	7
10	3	2	9	8	4	2	26	2	0
11	4	3	8	7	10	14	7	4	4
12	2	2	3	4	3	6	6	2	2
13	24	14	2	1	11	13	5	7	5
14	9	5	4	11	3	2	0	4	0
15	20	13	0	11	5	12	1	48	59
16	13	8	4	4	3	1	0	29	19
